# A Human-Centered Situation Security Framework Based on Multi-Sensor Data Fusion for Abnormal Behavior Recognition in the Industrial Internet

**DOI:** 10.3390/s26165090

**Published:** 2026-08-11

**Authors:** Kejing Zhao, Zhiyong Zhang

**Affiliations:** College of Information Engineering, Henan University of Science and Technology, Luoyang 471023, China; 220410040024@stu.haust.edu.cn

**Keywords:** industrial internet security, 5G sensor networks, multi-sensor data fusion, abnormal behavior recognition

## Abstract

To guarantee the robust security of the industrial internet, we propose a human-centered situational security framework that comprehensively considers multi-sensor industrial elements. Constructing an industrial spatial morphism mechanism allows for in-depth analysis of abnormal behaviors. Through the acquisition and effective analysis of multi-sensor data in industrial scenarios, a human-centered situation security is established, the computational complexity of the abnormal behavior identification model is reduced, while the accuracy and efficiency of identification are improved. This method has been verified for its generality in the processing data of metallic materials in 5G sensor networks. The experimental results demonstrate that the industrial situational triplets, validated through ISA-95 standard testing, achieve an average coverage rate of 99.6% in industrial scenarios. Extensive experiments on the Gas dataset and a real-world 5G-enabled production line demonstrated that the proposed method achieved mean precisions of 97.35% and 97.41%, respectively. Compared with the non-vectorized data, the processing time for 5G data and EdgeIIoT data decreased by 79.4 s and 52.9 s, respectively. The ablation study confirms that incorporating the intention component (*T*) yields a 13.92-percentage-point F1-score improvement over the *E* + *A* configuration. Through multi-dimensional performance validation, the security analysis framework for multi-sensor data in industrial scenarios enhances the identification performance of abnormal behaviors in the industrial internet and has excellent generalization capabilities.

## 1. Introduction

As Industry 4.0 has progressively deepened, industrial intelligence has gradually replaced traditional implementations of industrial expertise and frameworks while demonstrating superior performance in sectors such as energy [1], transportation [2], and health care [3]. The industrial internet represents a new type of infrastructure. Industry 4.0, characterized by the integration of cyber–physical systems, the Internet of Things, and cloud computing, provides the broader technological context for the industrial internet [4]. Within this paradigm, human–cyber–physical systems (HCPSs) have emerged as the core operational unit, where human operators interact with networked sensors and actuators. Ensuring the security of such HCPSs against evolving cyber threats constitutes the central problem addressed in this study. The security of sensor systems is of vital importance for the identification of abnormal behaviors in the industrial internet. With the development of the industrial internet, the volume of industrial data continues to grow, and cyberattack behaviors have become increasingly complex, not only deepening the threat level with predominant attack behaviors such as penetration attacks, backdoor attacks, and theft attacks but also posing significant security challenges to the network layer, application layer, and data layer of the industrial internet.

Working conditions refer to the operational status of equipment under conditions directly related to its actions, covering parameters such as temperature, pressure, and flow rate, as well as operational characteristics like control methods and load conditions. The industrial internet comprises a five-layer architecture [5], including a field layer that engages numerous devices, sensors, and actuators. The data generated in industrial scenarios, which typically includes operational data, sensor data, and production process data, are crucial components of the industrial internet and are commonly used for automation, monitoring, and predictive maintenance in industrial production. These data feature real-time, time series, and cyclical properties, accurately reflecting the operating states of the production equipment and efficiency fluctuations in the production line. As industrial internet data often exist in both structured and unstructured states [6], distinguishing and transforming these data leads to significant storage and computational costs. Traditional data characterization methods [7] primarily select the structural information of data to describe the dataset itself, which only adapts to binary datasets and has an overall poor performance. Additionally, some scholars use rule-based methods [8], but such approaches require experts to formulate rules, demand high completeness and compatibility, and have limited applicability [9].

Existing industrial anomaly detection methods exhibit three fundamental limitations. First, they treat human operators as exogenous noise rather than integral to the security loop, rendering them unable to detect attacks exploiting intention conflicts or behavioral anomalies. Second, current data representations cannot jointly encode observable physical events and unobservable cognitive states, such as malicious intent. Third, situation-aware frameworks have not been formally adapted to industrial CPSs with their multi-sensor heterogeneity and real-time constraints.

**Figure 1:**
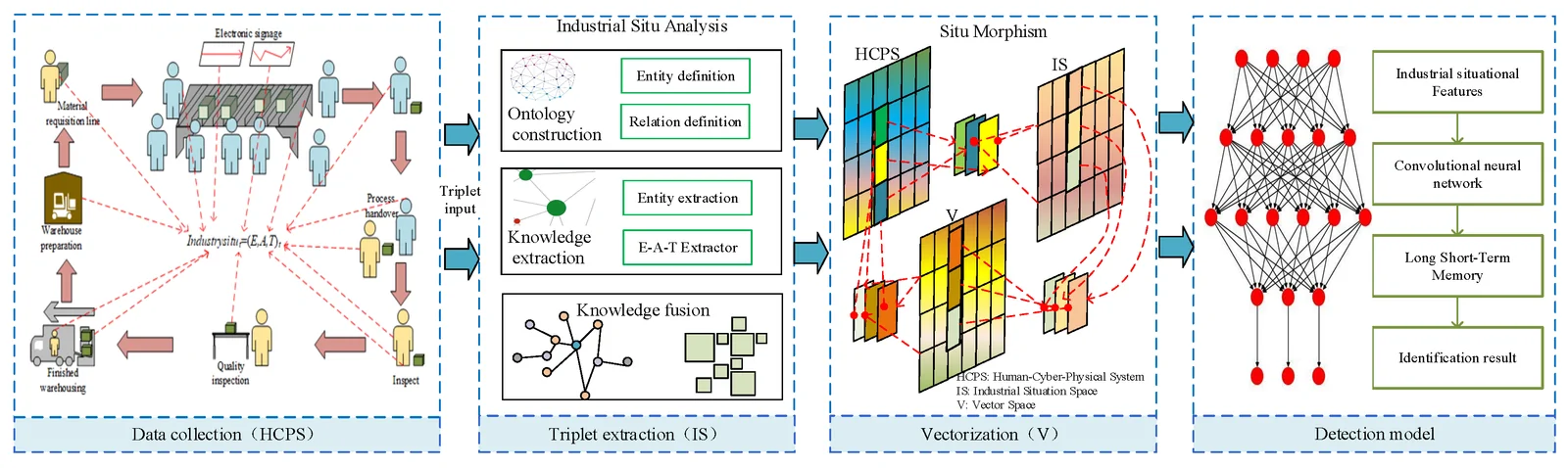
The overall architecture of the proposed human-centered situational security framework. The pipeline transforms raw HCPS data through four stages—collection, triplet extraction, morphism-based vectorization, and CNN-NLSTM classification—to produce anomaly detection results with component-level interpretability.

In view of this, this paper employs the fundamental concepts of situation analytics [10] and proposes the construction of industrial situation elements and the integration of industrial production data. By using a novel mechanism named industrial situation morphism, akin to the common vector morphism, industrial situation features are employed to achieve the high-precision detection and identification of industrial internet network attacks. The overall process is illustrated in Figure 1. This article focuses on two core issues, namely, formalizing and extracting the human-centered security elements, and efficiently vectorizing the triplets to support real-time anomaly detection. The main contributions of this paper are as follows.

1. We have proposed an overall framework for identifying abnormal behaviors in the industrial internet, established a human-centered situation security analysis framework, and laid the foundation for maintaining the security of the industrial internet.
2. To address the redundancy and inefficiency issues in industrial multi-source data processing, we have proposed an industrial situation morphism mechanism, which converts HCPS data into vector data, significantly enhancing operational efficiency and enabling the efficient analysis and real-time detection of industrial abnormal behaviors.
3. Sufficient experiments were carried out on open-source datasets, simulation platforms and a 5G sensor network intelligent production line. The results show that the proposed human-centered working condition security framework has high effectiveness, and the detection rate of abnormal behaviors can be increased to 99.96%. At the same time, the experiments show that this method demonstrates promising generalization capability within the evaluated scenarios.

This architecture consists of four stages: (1) HCPS data collection from multi-modal sensors; (2) situational triplet extraction, where raw data are mapped to (E, A, T) components; (3) situational morphism and vectorization via the covering morphism (π), which transforms the triplet space into a computable vector space while preserving structural similarity; (4) CNN-NLSTM-based anomaly detection, where the vectorized representation is fed into the hybrid network for binary or multi-class classification. All acronyms and symbols are defined in Section 3.

## 2. Related Work

To ensure the security of the industrial internet, effective anomaly detection mechanisms need to be established to reduce the harm caused by industrial production attacks. Current research on industrial anomaly behavior detection focuses mainly on constructing high-performance detection models and establishing effective industrial data representation schemes.

### 2.1. Data Representation Based on Machine Learning

High-performance anomaly detection models covering different industrial scenarios have been proposed to enhance industrial internet security. Focusing on attack detection in human–cyber–physical systems, the authors of [11] proposed a distinguishable reachability analysis-based attack detection method that identifies false data injection attacks through projection and space transformations. However, this approach assumes perfect knowledge of the system model and does not account for human-induced behavioral variations, limiting its applicability in dynamic industrial environments. A watermark signal-based detection method was proposed to detect replay attacks, and experiments proved its effectiveness. Knowing that invisible attacks in HCPSs cannot be easily detected via traditional methods, the authors of [12] proposed a detection model for replay and stealth attacks in discrete-event systems by integrating permutation matrices into the input signals, reporting that by disrupting the attacker’s concealment method, the precise detection of stealth attacks can be achieved. Nevertheless, the method relies on discrete-event abstractions that may not capture continuous physical dynamics, and human operator influences are entirely excluded from the detection framework. Driven by real data, a network attack detection method proposed by the authors of [13] simulates attacker behavior through reinforcement learning and uses a trained model for multi-attack category detection. Although reinforcement learning enables adaptive detection, the method treats attacks purely as statistical anomalies in sensor data, overlooking the semantic context of human intentions that often distinguishes malicious from benign operations. For zero-day attacks in industrial scenarios, the authors of [14] proposed a real-time malicious traffic detection system based on machine learning to achieve high performance and high throughput via frequency domain features. In view of the usefulness of real-time traffic data, the authors of [15] proposed XGRU-IDS, which is an interpretable hybrid type of deep learning IDS. It aggregates graphs and feature importance scores and strives to provide classification basis by category, and it has been evaluated on the CICIoT2023 dataset. While traffic-based methods offer real-time advantages, they operate exclusively at the cyber layer and provide no visibility into physical process anomalies or human behavioral deviations. The authors of [16] proposed a noise-resistant neural distillation framework BGA for encrypted threat intelligence. Its core lies in integrating the bidirectional long short-term memory network (BiLSTM) to extract time-dependent relationships, as well as the adaptive gated multi-head attention mechanism. Extensive evaluations on the CIC-IDS-2018 and Edge-IIoT benchmark datasets show that the performance upper limit on all key indicators exceeds 95.2%.

### 2.2. Data Representation Based on Graph Structure

One general belief is that an effective representation of industrial data can enable the extraction of hidden information in industrial production data, and the control over the entire industrial production process can be much enhanced. Starting from advanced feature selection to reveal hidden information, the authors of [17] propose a detection strategy based on graph autoencoders, which captures the spatiotemporal features of the power system, thereby providing improved detection performance. However, graph autoencoders treat the system as a purely physical process without modeling the human operators who interact with the power grid, leaving intention-driven attack vectors undetected. The authors of [18] proposed a DDoS attack detection scheme based on centralized federated learning. On this basis, the authors of [19] proposed a new binary particle swarm optimization wrapper framework for feature selection, which improves the intrusion detection accuracy of machine learning methods by enhancing the coupling between feature selection and training, effectively alleviating the redundancy problem in the current industrial data representations. The authors of [20] imitated the behavior of attackers to modify attack traffic data, making it easier to extract effective features without the need for additional knowledge, and the experiment results have proven its effectiveness. Since industrial attack data often exhibit high-dimensional characteristics, the authors of [21] proposed a higher-order network embedding framework, introducing a universal diffusion mechanism for the subgraph node embedding of node neighborhoods to significantly improve industrial attack detection. However, it does not address the representation of human factors, as the graph structure captures only device-level interactions.

In summary, existing industrial anomaly detection methods suffer from three fundamental limitations. First, they predominantly treat human operators as exogenous factors or noise rather than as integral components of the security loop; consequently, they cannot detect attacks that exploit human intention conflicts or behavioral anomalies. Second, current data representations lack the structural capacity to simultaneously encode observable physical events and unobservable cognitive states. Third, while situation-aware frameworks have been proposed in general contexts, they have not been formally adapted to industrial cyber–physical systems with their unique multi-sensor heterogeneity and real-time constraints. Table 1 summarizes the key limitations of representative studies. In view of this, this paper proposes constructing an industrial situation space with industrial situation triplets to characterize real-time industrial scenes with the hidden, unobservable yet inferable human mental states often neglected in other anomaly detection models. We then present the industrial situation morphism mechanism, described as the transformation from the industrial situation space to the vector space. This approach establishes the computability of the industrial situation space and completes the real-time detection and identification of industrial internet anomalies on this basis.

## 3. Related Definition

**Definition 1** (Situation Data)**.**
*This is a collection of all industrial scenario elements within a certain period. Situation data includes two specific subsets: η and μ. H contains all the irrelevant or noisy data in industrial scenarios. μ contains all the observable and unobservable data in the industrial production process. Notably, η and μ do not necessarily overlap.*

**Definition 2** (Industrial Situation)**.**
*The industrial situation is defined as a triplet industrysitu_t_ = (E,A,T), where E represents the industrial environment, including the temporal and spatial dimensions, which usually includes data obtained by sensors, such as temperature and humidity, vibration, and current in the industrial site. A corresponds to actual observable behaviors in industrial scenarios, such as operations, inspections, scheduling, and attack actions, which are obtained through the action equipment sensors. T represents the target of abnormal behavior, and it can be expressed as <en, ob, d>_t_, where en represents entity information, such as biological information, ob represents the objective of operational behavior, attack, etc., and d represents the inferred intention [9,10] for industrial control behavior, such as hidden attempts to manipulate [22]. In practice, the target tuple (T) comprises three categories of information: directly observable, manually annotated, and inferred. Directly observable information encompasses explicit operational instructions, standardized job identifiers, and biometric authentication records. Manually annotated information consists primarily of historical attack labels extracted from security event logs. Inferred information represents latent target signals deduced from discrepancies between observed user behaviors and predefined operational objectives. The framework proposed in this paper mitigates uncertainty in the inferred component by introducing a probabilistic target strength scoring mechanism. Taking industrial production safety as an example, when the firewall alarms (E) and realizes that an attack has occurred, it is best to protect the system immediately (T), so the corresponding port is closed (A).*

**Definition 3** (Industrial Situation Space Lattice (Z) and Covering Morphism (π))**.**
*The industrial situational space is formalized as a lattice structure (Z = E × A × T), where E means Environment Lattice, and the element is the device–network–physical status tuples e ∈ D × N × P; A means Action Lattice, and the element is the behavior sequence a ∈ {0,1}^m^; T means Target, represented as* $T\in {\mathbb{R}}^{d}$ *. When operators exhibit intention conflicts, a dynamic conflict intention handling method is established to balance safety and efficiency considerations in the context of industrial production:*

(1) $$\underset{m\in M}{Max}\left[w\cdot safety\left(m\right)+\left(1-w\right)\cdot efficiency\left(m\right)\right]$$

*where w = sigmoid(risk(e)).*

*The covering morphism (π) is defined as follows:*

(2) $$\pi \left(S\right)=\left(\prod_{e_{k}\in E_{S}}e_{k},\underset{a_{j}\in A_{S}}{\cap }a_{j},\underset{t_{i}\in T_{S}}{\cup }t_{i}\right)$$

*where* ∏ *signifies the environmental tensor product quantity; ∩ denotes the intersection operation of behaviors, preserving the common support set; ∪ represents the union operation of intentions, taking the maximum intention strength.*

**Definition 4** (Industrial Situation Sessions and Industrial Space)**.**
*Industrial situation sessions are sequences of industrial scenarios arranged in sequence within a specific time frame to achieve production objectives. Industrial space includes m situational sessions, each encompassing n industrial scenario sequences:*

(3) $$Industrial\;Space=\left\{\begin{array}{c} IS_{11},IS_{12},IS_{13},IS_{14},\ldots ,IS_{1l}\\ \ldots \ldots \\ IS_{m1},IS_{m2},IS_{m3},IS_{m4},\ldots ,IS_{mn} \end{array}\right\}$$

### Mathematical Properties of Industrial Situational Space Lattice and Bounded Information Loss Theorem

Let the situational space be *Z* = *E* × *A* × *T*. For any $S_{1}=\left(e_{1},a_{1},t_{1}\right)$ and $S_{2}=\left(e_{2},a_{2},t_{2}\right)$, define a partial order ($\underline{≺}$) such that $S_{1}\underline{≺}S_{2}\Leftrightarrow e_{1}\subseteq e_{2}$ and $a_{1}\subseteq a_{2}$ and $t_{1}\subseteq t_{2}$. Then,

1. Z forms a complete distributive lattice.
2. For the covering morphism (π), *Z* → *V*, where V is the vector space. And for its reconstruction (π^−1^), the information loss ($l_{map}=\left\|S-\right.\pi^{-1}{\left.\left(\pi (S)\right)\right\|}_{F}^{2}$) is strictly bounded by the truncated singular values of the intention matrix.

**Proof.**  For any non-empty subset ($S={\left\{\left(e_{i},a_{i},t_{i}\right)\right\}}_{i\in I}\subset Z$), define its infimum and supremum, respectively, as per (4):

(4) $$\inf\left(S\right)=\left(\underset{i\in I}{\cap }e_{i},\underset{i\in I}{\cap }a_{i},\underset{i\in I}{\inf t_{i}}\right),\sup\left(S\right)=\left(\underset{i\in I}{\cup }e_{i},\underset{i\in I}{\cup }a_{i},\underset{i\in I}{\sup t_{i}}\right)$$

The intersection and union operations are closed in their respective domains, and the distributive law holds. Thus, every subset has a unique least upper bound and greatest lower bound, proving 1.

For 2, the intention component (*T*) embeds as a real-valued matrix ($M\in R^{d\times n}$). The vectorization morphism (π) performs a truncated singular value decomposition (SVD) on *M*, retaining only the top-*k* singular values, where $k=\dim\left(V_{T}\right)$. Let the full SVD be *M* = *U∑V^T^* with ∑ = *diag*(*σ*_1_, *σ*_2_, …, *σ_T_*), and *σ*_1_ ≥ … > *σ_T_*. The low-rank approximation after morphism is *M_k_* = *U_k_∑_k_V_k_^T^*. By the Eckart–Young–Mirsky theorem, the optimal rank-*k* approximation error in the Frobenius norm is exactly ${\left\|M-\left.M_{k}\right\|\right.}_{F}^{2}=\sum_{i=k+1}^{T}\sigma_{i}^{2}$. The encoding of *E* and *A* introduces only negligible quantization noise (*ε*). Hence, the total mapping loss is bounded by $l_{map}\leq \sum_{i=k+1}^{T}\sigma_{i}^{2}+\varepsilon$. Since attack intentions in industrial data typically concentrate in the first few principal singular directions, choosing *k* to preserve >99% cumulative energy ensures $\sum_{i=k+1}^{T}\sigma_{i}^{2}\to 0$, which theoretically justifies the near-zero empirical loss observed in our experiments. □

Table 2 shows summary of principal mathematical symbols. 

## 4. Proposed Method

Based on the triplet structure defined in Definition 2, we constructed industrial situation data on the actual data. The covering morphism in Definition 3 provides a structure-preserving guarantee for vectorization, ensuring that the similarity relationship in the vector space is consistent with the original situation space. This property enables the subsequent convolutional neural network–nested long short-term memory (CNN-NLSTM) model to achieve efficient computation while retaining the interaction of human factors. The NLSTM model updates the cell state by embedding LSTM units, thereby enhancing the model’s operational efficiency [23]. In an industrial internet scene, the industrial space perfectly depicts industrial production by symbolizing the actual human–cyber–physical scenario, as well as all the operational processes it covers, which encompasses a vast amount of industrial information. Due to the high-dimensional heterogeneity of industrial production data, the performance of current industrial internet anomaly identification is often limited by the input of the model. In this paper, industrial situation triplets are constructed to formally represent industrial feature data, and industrial situational morphism is taken as the core to achieve the vectorization of industrial data, aiming to improve the accuracy and efficiency of industrial internet anomaly attack identification. Numerous experiments have verified that this method has a good performance in identifying complex attacks on the industrial internet, and the demonstration of its promising generalization capability within the evaluated scenarios also provides a basis for its application in other industrial scenarios. This section, which is based on the construction of an industrial situation triplet, provides a detailed introduction to industrial situation data representation and the industrial situation morphism mechanism.

Based on the framework structure shown in Figure 1, first, industrial site data is collected from multiple sensors. Next, based on the industrial process, industrial situation triplets are extracted in a complete production process. Then, through situation morphism, the situation data is mapped into more computable vector data. Finally, the data in the vector space is used as the input of the CNN-NLSTM detection model, and the analysis and identification of attack behaviors in the industrial scene are output.

### 4.1. Industrial Situation and Situation Data Representation

Within the industrial internet and its encompassing human–cyber–physical space, industrial entities, including human–cyber–physical entities, contain massive, heterogeneous, and discrete industrial situation features, such as digital identities, spatiotemporal environments, and behavioral operations. Taking common application scenarios of the industrial internet as research objects, common information of industrial control objects, such as the equipment in industrial production and identity information such as workers’ job numbers, is not enough for a comprehensive representation of the industrial internet; it must be combined with the mental state of the current (and pretended) workers. Considering a complete industrial production cycle, three elements of human-centered industrial situation analysis are extracted: environment (*E*), behavior or action (*A*), and the target state (*T*). *T* usually represents both observable and hidden behavioral target states, such as the targets targeted by attack behaviors. Thus, the actual data of concern to be extracted from industrial human–cyber–physical systems are such triplets. Unless necessary, in the remainder of the paper, we will mostly refer to situational data as triplets (i.e., *E*, *A*, *T*), as explained before.

The industrial internet is closely related to intelligent manufacturing, and the human–cyber–physical system characteristics centered on humans exhibit significant heterogeneity, with continuous interactions among components within human–cyber–physical systems. Industrial space data primarily consist of time series data, and because of the different sources of data, it is difficult to form a complete description of the entire industrial scene. This presents significant obstacles to the practical application of collected industrial data [23]. In view of such difficulties, this paper combines industrial situation and human-centered human–cyber–physical system data representations and an inferring morphism mechanism between the human–cyber–physical space, industrial situation space, and vector space.

### 4.2. Industrial Situation Morphism Mechanism

A morphism is a structure-preserving function that morphs from one structure to another, abstracting a process that maintains the structure between two mathematical structures; namely, it is a “structure-preserving” morphism. Morphisms are used in sets, groups, fields, and spaces and exist in various forms, such as functions, group homomorphisms, continuous functions, and homomorphisms. The function *f* is a morphism from space A to space B if and only if $if\;\left(x,y\right)\in A\Rightarrow \left(f\left(x\right),f\left(y\right)\right)\in B$ holds [24]. Morphism functions have composition and identity operations. Assuming that *f* is a morphism from any space A to space B, (5) is valid:

(5) $$\forall m,n\in A,f\left(m+n\right)=f\left(m\right)+f\left(n\right)\;and\;f\left(am\right)=af\left(m\right)are\;true$$

The goal of situational morphism is to convert situational triplets into vectors to facilitate the unified form and normalization of industrial data. Since finding the nearest neighborhood involves calculating the similarity between situations, this paper selects similarity as the main structure to be preserved in situational morphism. In response to the significant features of attack behaviors, such as temporal and spatial correlations, which are based on the morphism function from the industrial situational space (*IS*) to the vector space (*V*) in the industrial situation morphism computation, (6) is provided:

(6) $$g\left(IS\to V\right)$$

When $\forall is_{a},is_{b}\in IS,sim\left(is_{a},is_{b}\right)>\delta$, then $sim\left(g\left(is_{a}\right),g\left(is_{b}\right)\right)>\delta$, where 0<δ<1. Specifically, g is a function whose domain and range are both situation data, i.e., g: situation data → situation data.

Existence and Uniqueness of Industrial Situation Triplets. Any industrial scenario (S∈ζ) can be mapped to a unique triplet ((*E*,*A*,*T*) ∈ *Z*). First, define the feature function (*h*) (7):

(7) $$h(S)=[\alpha \cdot \mathrm{rank}(m),\beta \cdot \mathrm{entropy}(a),\mu \cdot \left\|e\right\|]$$

where *rank*(*m*) denotes the rank of the intent vector, *entropy*(*a*) denotes the entropy of the action sequence, and $\left\|e\right\|$ denotes the Euclidean norm of the environmental state. The encoding function (*h*(*S*)) is designed as a practical vectorization strategy rather than a strictly injective mathematical mapping. The three components are chosen to capture complementary aspects of the situational state. While injectivity is not guaranteed in the strict mathematical sense, the empirical evaluation in Section 5 demonstrates that the resulting representation preserves sufficient discriminative information for anomaly detection tasks, with the practical information loss bounded as derived in properties of industrial situational space lattice and bounded information loss theorem. The vector (v) obtained through morphism is directly used as the input tensor for the CNN-NLSTM, with each dimension corresponding to the encoded *E*, *A*, and *T* features. Table 3 summarizes the complete morphism process from the HCPS to the IS and then to the V, as well as its industrial significance.

### 4.3. Anomaly Recognition Model

**Figure 2:**
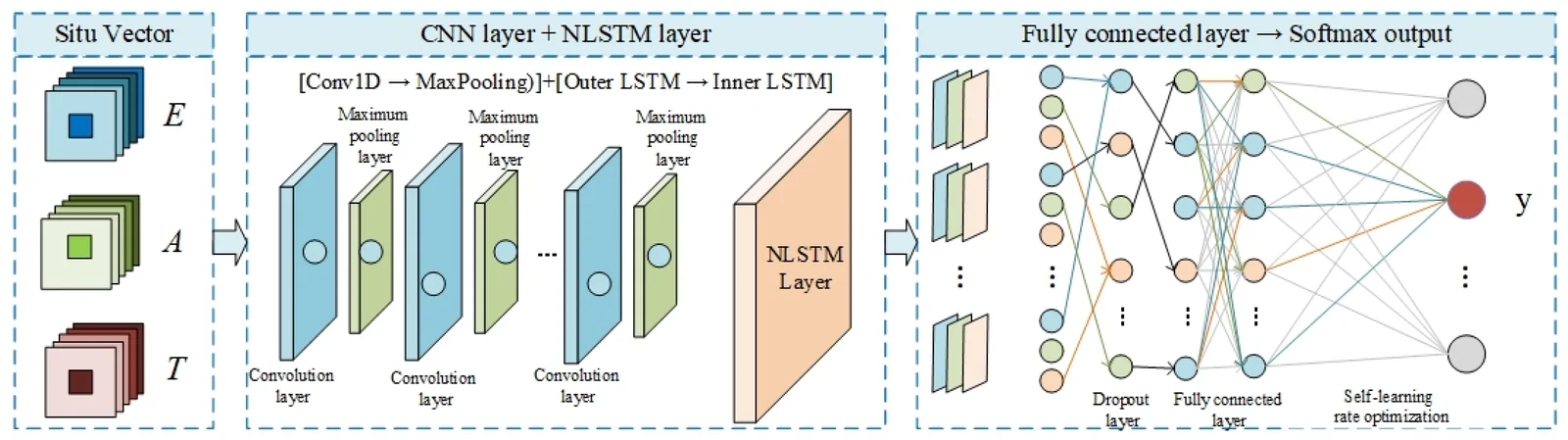
CNN-NLSTM detection model structure chart.

To further achieve efficient analysis of industrial abnormal behaviors, we construct the CNN-NLSTM model based on the traditional LSTM. CNNs excel at extracting high-level abstract features from grid-like data. Taking contextual data as the input of the CNN-NLSTM hybrid model can further improve the performance of attack behavior recognition. Human-centered situation data generalizes industrial scenarios at any given moment by constructing triplets, adding human factors, and centering on the environment in which people are located, the actions they take, and the intentions behind them. The situation data, after industrial situation morphism, is used as the input of the model, which further enhances the computational efficiency and the performance of identifying attack anomalies. The embedded neuron is ${\tilde{C}}_{t}=m_{t}\left(f_{t}\cdot c_{t-1},i_{t}\cdot g_{t}\right)$. The structure of the hybrid neural network model is shown in Figure 2.

The CNN-NLSTM architecture comprises: Convolutional Layers: two 1D convolutional layers with 96 and 128 filters, kernel sizes 3 and 5, respectively, each followed by *ReLU* activation and max-pooling; NLSTM Layers: two stacked NLSTM layers with 256 units each, using the nested cell structure described in [25] with the output dimension of the inner LSTM set to 64; Dropout: 0.5 applied after each NLSTM layer; Output: a fully connected layer with softmax activation for classification. Training uses the Adam optimizer with a learning rate of 0.001, a batch size of 64, and early stopping based on validation loss.

## 5. Experiment

### 5.1. Experimental Data

To further validate that the industrial situation triplets can comprehensively cover all industrial scenarios, this study randomly selected 100 scenarios (spanning Levels 0–4) from the ISA-95 standard [26] to verify the coverage morphism $\pi \left(S_{i}\right)\neq Ø$.

According to the ISA-95 standard, industrial systems are hierarchically structured into five levels: Level 0: the physical device layer; Level 1: the process control layer; Level 2: the monitoring and operations layer; Level 3: the production management layer; Level 4: the enterprise resource planning (ERP) layer. Based on the standard documentation and practical production experience, 30 initial scenario pools were constructed for each level. A stratified random sampling approach was then applied, selecting 20 scenarios per level (totaling 100 scenarios) for coverage validation. If the scenario pool for a specific level contained fewer than 30 scenarios, repeated sampling was permitted, with such cases explicitly labeled as “duplicate scenarios”. The results are shown in Table 4. This methodology ensures robust validation across all hierarchical layers while maintaining statistical rigor. Although the ISA-95 standard does not inherently include an independent human factor database, its process descriptions at Level 2 (monitoring and operation layer) and Level 3 (production management layer) involve operator behaviors such as monitoring, scheduling, and emergency response. From these descriptions, we extract the corresponding action sequences and intent labels to construct E-A-T triplets.

Analysis of Uncovered ISA-95 Scenarios. Although the coverage rate reaches 99.6% on average, the stratified sampling reveals that Level 2 (monitoring and operations) and Level 4 (enterprise resource planning) exhibit a coverage rate of 99%, with one unique scenario missing in each layer. The missing scenarios are as follows: (1) Level-2 Operator Intention Conflict—a situation where two operators issue contradictory control commands (e.g., emergency stop vs. speed-up) to the same actuator under time-critical conditions—and (2) Level-4 Cross-Enterprise Abnormal Order Interference—an unexpected drastic fluctuation in external supply-chain orders that disrupts the prescheduled production rhythm.

The key point is that the static ISA-95 document lacks these two specific cases, which does not mean that our situation model is flawed. For these unobserved dynamic semantic scenarios, we adopted a boundary intention softening strategy in the preprocessing stage, mapping the conflict intensity to a moderately strong virtual *T* vector. Therefore, the model trained on the remaining scenarios can still maintain robust reasoning ability for these unobserved situations because the underlying spatiotemporal features of the environment (*E*) and action (*A*) are fully retained.

Raw sensor data are synchronized using the production-line timestamp and then segmented into non-overlapping windows of 30 s. Missing values are imputed using linear interpolation. Each window is normalized via min–max scaling, with the scaling parameters fitted exclusively on the training split to avoid data leakage.

#### 5.1.1. Experimental Environment

Many experiments have been conducted to verify the significant role of industrial situation elements in improving the performance of industrial internet anomaly detection. The hardware configuration for the experimental environment includes a 12th Gen Intel(R) Core(TM) i5-12500 processor, 16 GB of RAM, and a 64-bit Windows 11 operating system. The software environment uses the Python 3.6 language. This paper selects the accuracy, precision, FA, FNR, recall, and F1-score as the metrics to evaluate attack detection performance, with the specific calculation methods listed as (8):

(8) $$\begin{array}{l} Accuracy=\frac{TP+TN}{TP+TN+FP+FN}\\ Precision=Detection\;Rate\left(DR\right)\\ Recall=\frac{TP}{TP+FN}\\ F1-Score=2\frac{Precision*Recall}{Precision+Recall} \end{array}$$

#### 5.1.2. Data Collection and Preprocessing

**Figure 3:**
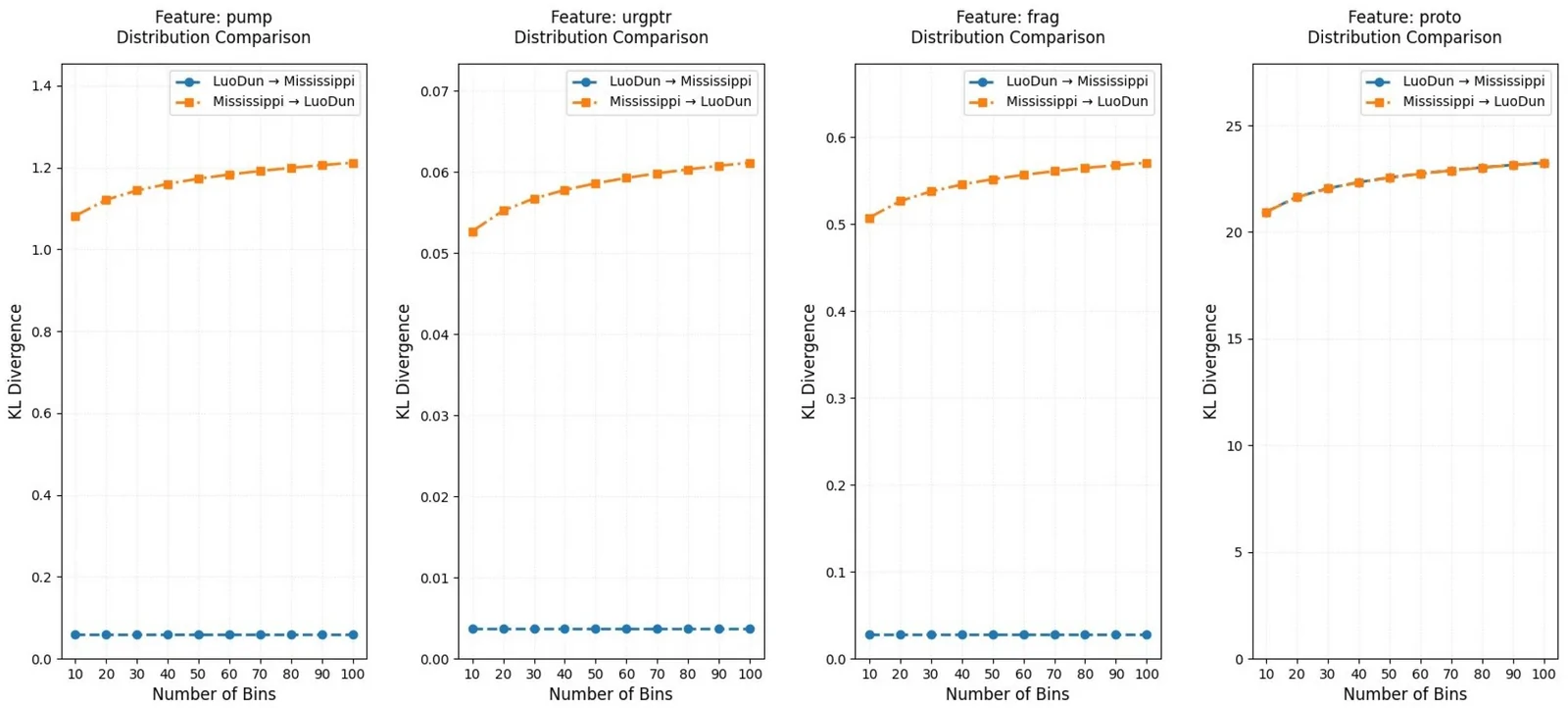
KL divergence between LuoDun and Gas datasets.

This study selected the online industrial internet security platform LuoDun (http://www.luodun.cloud/) for training environment modeling (non-attack scenarios) while employing natural gas pipeline security standard data (Gas) from Mississippi State University to validate attack detection. The LuoDun platform comprises client-side and server-side data, functioning as an online industrial internet security platform that collects information through sensors deployed in actual industrial production processes. The collected industrial data is subsequently visualized as tabular or graphical representations on the platform. The Gas dataset collected by Mississippi State University contains over 60,000 records with 18 features and seven attack categories: NMR, CMRI, MSCI, MPCI, MFCI, DoS, and Reconnaissance. KL divergence between LuoDun and Gas datasets are shown in Figure 3.

To validate the comparability of feature distributions between the normal-operating-condition data collected by the LuoDun platform and the Gas attack dataset, we computed the Kullback–Leibler (KL) divergence for each shared feature. As the number of bins increases, the bidirectional KL divergence stabilizes below 0.05, indicating a high degree of distributional similarity between the two datasets’ shared features, which supports the feasibility of combining LuoDun normal samples with Gas attack samples for cross-domain validation in their numerical distributions. Consequently, Gas attack samples can be injected into the LuoDun baseline for mixed training.

To mitigate the impact of multi-source data fusion on the experimental results, min–max normalization was applied for data standardization. Additionally, the Synthetic Minority Over-sampling Technique (SMOTE) was implemented to address data imbalance issues. Importantly, all preprocessing operations were applied exclusively to the training data after the train/validation/test split, ensuring that no information from the validation or test sets influenced model training.

To further validate the generalization capability of the proposed framework in real industrial settings, we constructed a dedicated dataset from an intelligent non-ferrous metal-processing production line equipped with 5G sensor networks. This production line integrates multi-modal sensors, including temperature, high-frequency vibration, three-phase current, acoustic emission, and 5G network quality metrics. The data were continuously collected from 10 June 2021, to 22 October 2021, covering normal operations and several manually injected fault/attack scenarios. Table 3 summarizes the statistical profile of this dataset. The heterogeneous nature poses significant challenges for unified feature extraction, precisely motivating the need for our situational triplet abstraction and morphism mechanism. A statistical profile of the 5G non-ferrous metal production dataset is shown in Table 5.

### 5.2. Industrial Binary Classification Anomaly Detection

**Figure 4:**
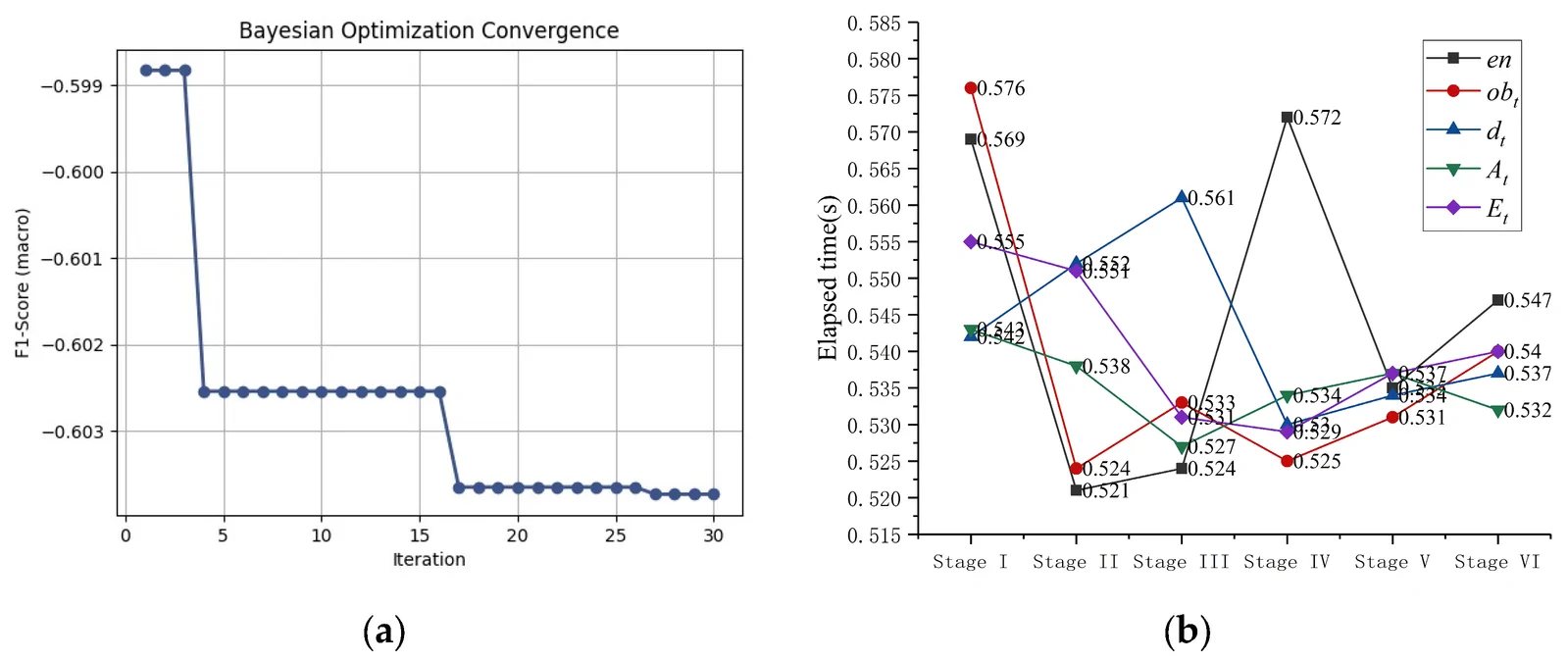
(**a**) Bayesian optimization coverage testing; (**b**) time complexity of industrial situation morphism.

Owing to the presence of large amounts of heterogeneous data in actual industrial settings, it is necessary to extract key features when identifying anomalies. Therefore, this paper proposes a deep neural network attack detection model—a hybrid convolutional neural network and nested long short-term memory model (CNN-NLSTM). To further enhance the model performance, we employed the Bayesian optimization method, using the validation set’s F1-score as the objective function for hyperparameter tuning. The key optimized parameters included the Learning-rate, NLSTM-units, Dropout-rate, and Conv-filters. The experimental results are presented in Figure 4a and Table 6.

First, incorporating industrial situation features into the anomaly detection model requires vectorizing the industrial situation data. The industrial situation morphism mechanism maximally retains the original information of the data while preserving the structure between the data points and morphs the data from the industrial situation space to a computable vector space. To verify the effectiveness of industrial situation elements in industrial anomaly detection, this paper first preprocessed the collected industrial situation triplet dataset collected based on situation data. Then, according to situation morphism mechanism, the industrial situation data were vectorized. The experimental results are shown in Table 7.

To ensure the reproducibility and statistical robustness of our experimental results, all reported metrics are computed as the mean over 10 independent training runs with different random seeds (42, 123, 456, 789, 1010, 1212, 1313, 1414, 1515, 1616). For each metric, we also calculate the standard deviation across these 10 runs. Notably, the standard deviations for all primary metrics are consistently below 0.03, indicating that our model converges stably and is insensitive to random initialization. This exceptionally low variance validates the reliability of the reported performance.

**Figure 5:**
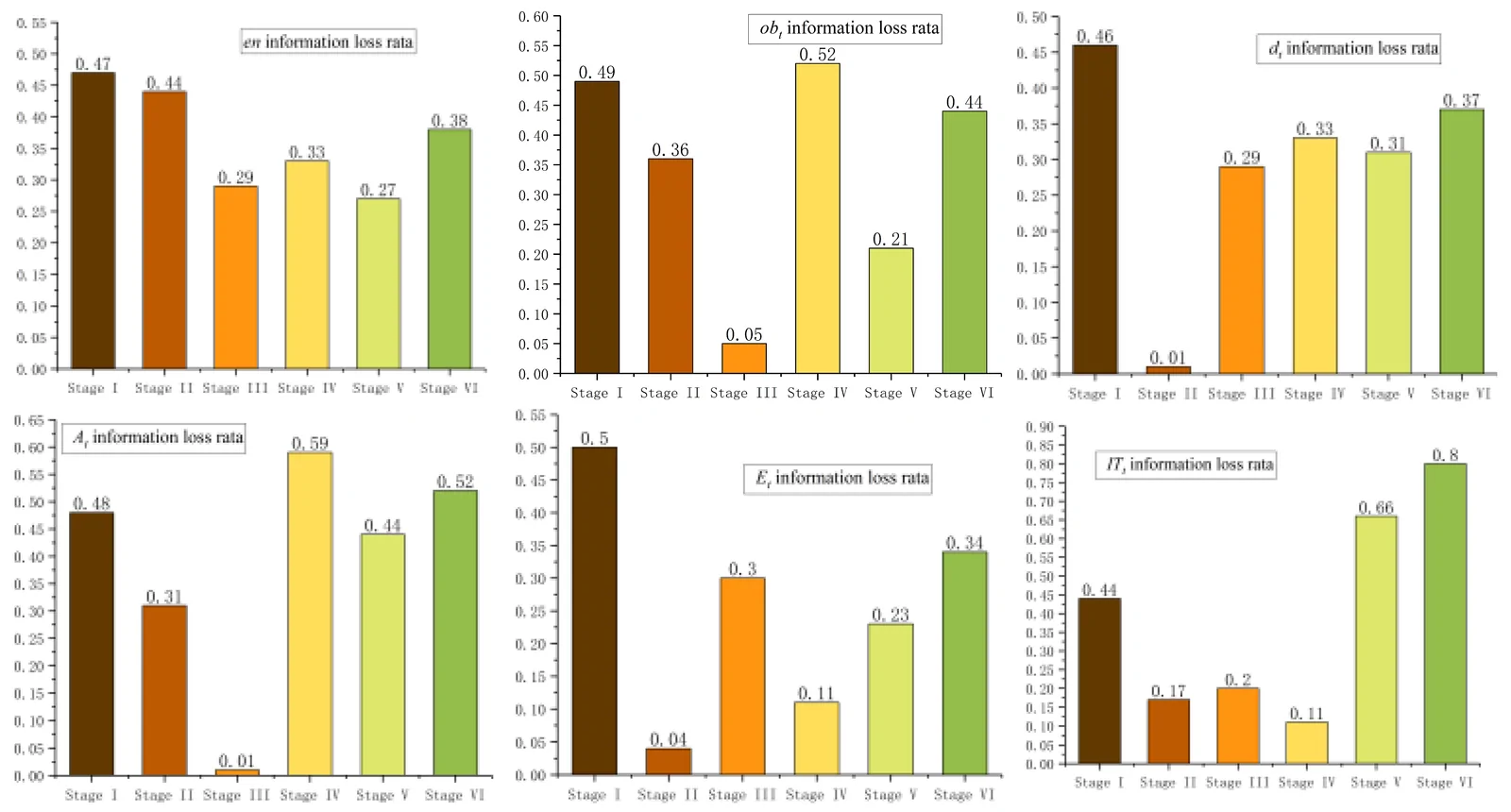
Industrial situation morphism information loss.

Industrial situation morphism aims to vectorize industrial situation data to identify anomalies efficiently and accurately in the actual production process. We convert the industrial scenario data into vector data through situation morphism, and we verify the effectiveness of the morphism method by calculating the information loss of each element during the morphism process. As shown in Figure 5, the information loss in both the first and sixth stages is relatively high, with an average value of 0.5. Moreover, the morphism information loss of entity information in the triplets of industrial situation is not very different at each stage, basically floating between 0.3 and 0.4, and the information loss of the environmental feature tuples always maintains a stable and ideal level throughout the whole industrial production process. As shown in Figure 4b, the time consumption of triplets varies at different stages. In stage I, the overall time consumption is relatively high, while in stage V, it is at a relatively low level. In short, the overall time consumption of triplets in industrial scenarios is below 0.58 s, meeting the time consumption requirements in actual industrial scenarios. With reference to the upper bound of delay tolerance for real-time anomaly detection in the industrial process control domain (typically 1 s), the triplet conversion time across all stages in this experiment was below 0.58 s, satisfying the real-time requirements for online deployment.

This empirical result aligns with the theoretical bound established in properties of industrial situational space lattice and bounded information loss theorem, where the truncated singular values of the high-dimensional industrial intention matrix decay rapidly, rendering the morphism loss numerically negligible.

### 5.3. Industrial Multi-Classification Anomaly Detection

To further verify the effectiveness of the proposed method, the Gas data and Vectorized Gas (Gas after situation morphism) data were simultaneously used as the input of the model for subsequent verification. Our research was also compared with existing research models [27,28,29,30,31] and the classical models. All the baseline models used as reference models were replicated using the same training data division, preprocessing, balancing, and optimization methods. The experimental results are shown in Table 8 and Table 9. The time consumption refers to the total time taken from end to end, including model prediction, inference, etc.

**Figure 6:**
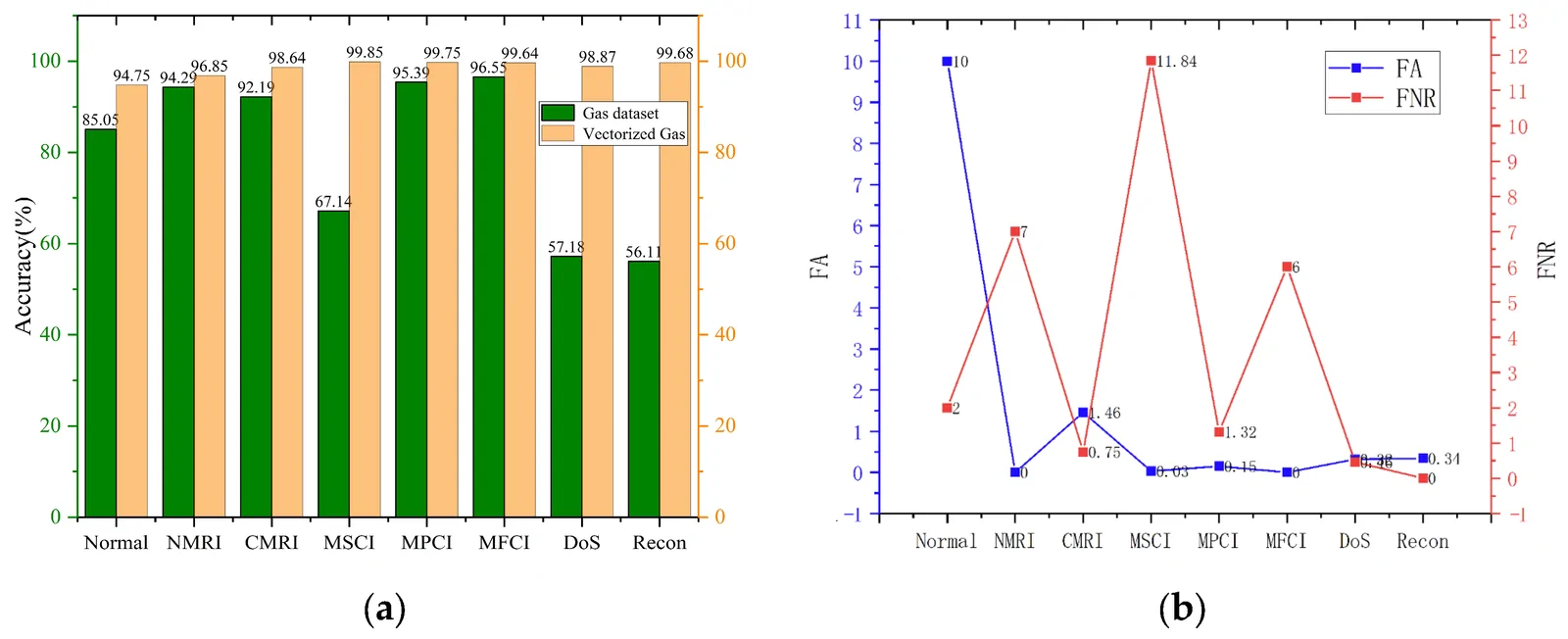
(**a**) Comparison of accuracies of different attack categories; (**b**) FA and FNR detected by different attack categories.

**Figure 7:**
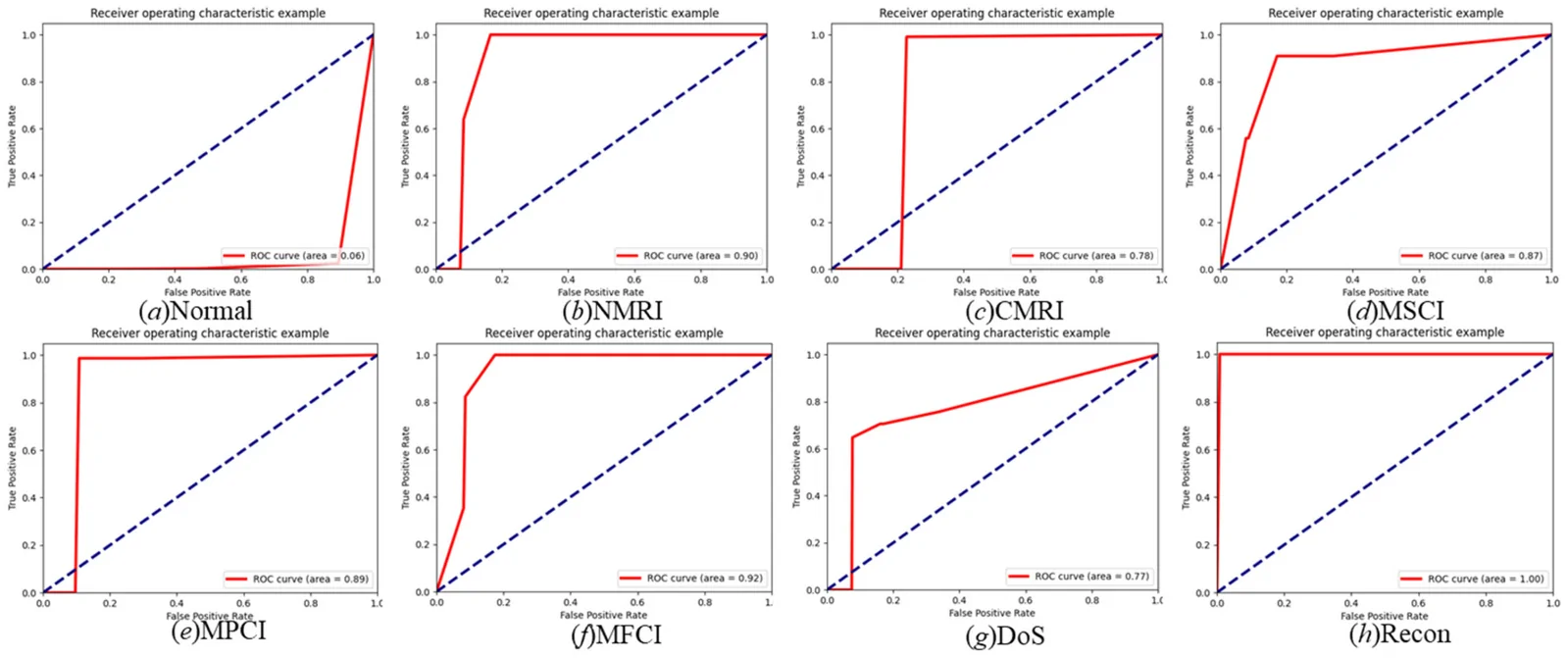
Comparison of ROC curves of different attack categories.

**Figure 8:**
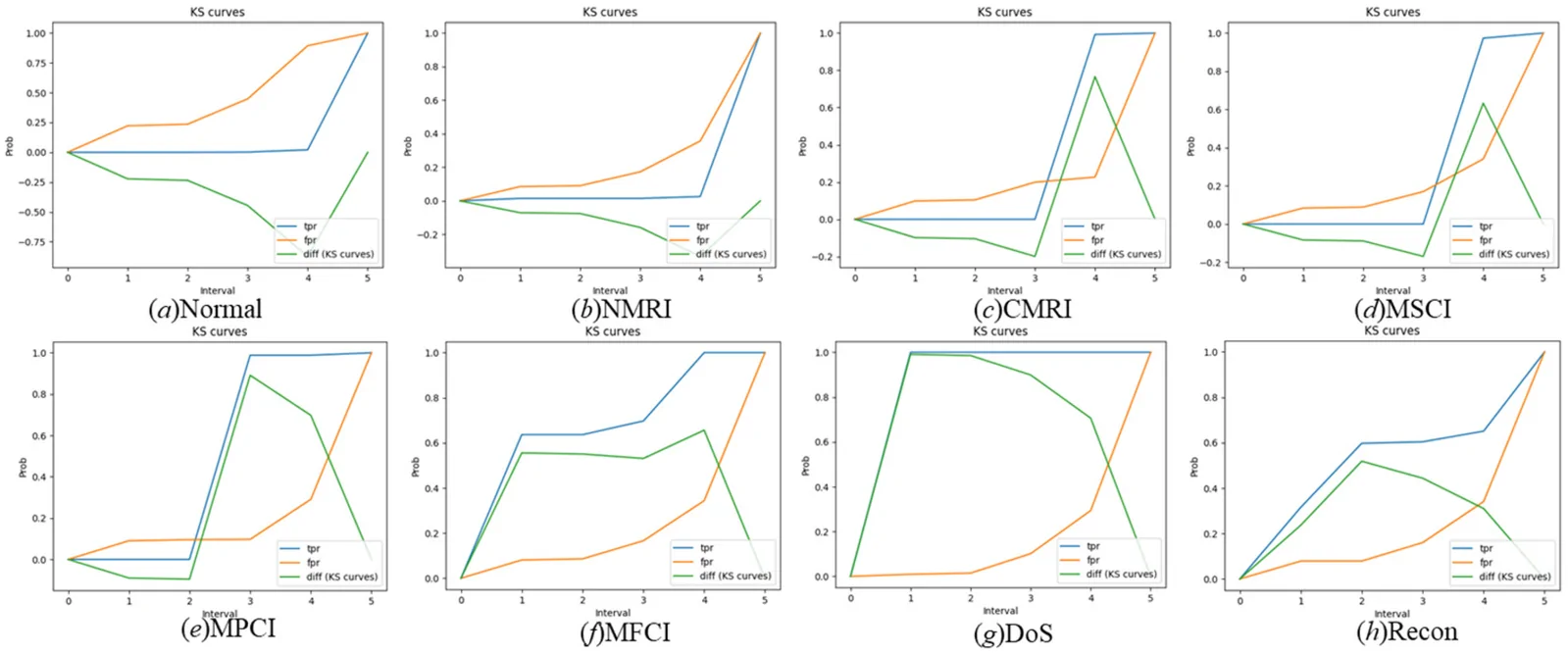
Comparison of KS curves of different attack categories.

As shown in Figure 6a, in Gas data that integrate elements from industry, the detection performance of eight attack categories was significantly improved compared with that of Gas data, among which the detection accuracies of the MSCI, DoS, and Recon attacks increased by more than 30%, and the recognition accuracies of other categories also increased to varying degrees. Moreover, the proposed method achieved particularly high detection accuracies for MPCI and MFCI attacks, reaching 99.85% and 99.75%, respectively. Figure 6b shows that the false-positive rates of NMRI, NPCI, and Recon attacks decreased significantly under the support of industrial situation elements, indicating that industrial situation elements have obvious advantages in describing industrial scenarios. Our model has the best detection performance against CMRI attacks, MPCI attacks, DoS attacks, and Recon attacks. According to Figure 7 and Figure 8, the ROC curve results are also different for the eight different attack categories, in which the ROC area of Recon attack identification is 1, the identification accuracy performance is significant, and the KS statistical curve is consistent with the ROC curve results, indicating that the situation elements in this paper strongly support anomaly recognition in the industrial internet.

Addressing the issue of data imbalance, this paper adopts a method combining SMOTE and Focal Loss to alleviate the problem wherein the recall rates of NMRI and NMCI are both 0. The experimental results are shown in Table 10.

The recall rate and F1-score of the model based on only SMOTE for the NMRI and NMCI attack categories were both 0 in the original data, indicating that the data imbalance was extremely severe. After optimization with Focal Loss, the detection performance of these two attack categories improved, but it still did not reach the ideal level. After combining SMOTE with Focal Loss, the detection performance of both methods was significantly improved, indicating that the data imbalance of NMRI and NMCI attacks was significantly alleviated.

By gradually adding the elements of triplets, we explored the influence of industrial situation triplets on the performance of industrial anomaly behavior detection. The experimental results are shown in Table 11. The results of the ablation experiments show that as the situation elements are gradually added, the F1-score of the model significantly increases, and the time consumption and GPU memory usage do not increase significantly, which can meet the requirements of actual industrial scenarios. The ablation study demonstrates a clear monotonic improvement as situational elements are progressively incorporated. The F1-score increase from 63.45 (using only E) to 97.35 (using E-A-T) is confirmed to be statistically significant via a paired t-test (*p* < 0.01), validating that each added dimension contributes non-trivially to anomaly detection performance.

### 5.4. Model Generalization Test

To further verify the application of the method proposed in this paper in actual industrial environments, the data were collected from the processing of metallic materials in a 5G sensor network intelligent production line [32]. Among the data, the categories were “attack” and “normal”. We also selected EdgeIIoT data as an experimental content for generalization verification. EdgeIIoT is an open dataset specifically designed for the Internet of Things (IoT) and edge computing environments. This dataset simulates typical IoT and edge computing scenarios and contains abundant traffic data, device behavior logs, and attack patterns. The data comes from various IoT devices, and the data features include alerts, system resources, logs, network traffic, etc. This dataset analyzes 14 attacks related to IoT and IIoT connection protocols and is suitable for model robustness verification in the IIoT environment. The binary classification experimental results obtained through data characterization and anomaly identification verification are shown in Table 12.

In the generalization experiment, when the vectorized data after industrial situation morphism was used as the model input, the overall performance of the model improved significantly compared to the original data. In the vectorized data experiment, the accuracy increased by 1.9%, and the time-consuming part of the model improved by 79.4 s, which is of great significance in actual industrial production. In the EdgeIIoT data experiment, the F1-score increased by 18.62%, and the processing time increased by 52.9 s. Through re-verification in the production data of non-ferrous metal processing, it is proved that the industrial situation morphism method proposed in this paper has certain generalization ability.

## 6. Discussion and Future Directions

In order to address the issues of the high time consumption and low performance of anomaly behavior analysis models in industrial internet scenarios, this paper proposes a conceptual triplet representation of industrial situations, which improves the performance of the abnormal behavior detection model in industrial scenarios through a situation security analysis method. By integrating multi-sensor data, the proposed framework significantly enhances security systems driven by sensors, further ensuring the security of the industrial internet. Further verification through the actual industrial data of 5G sensor networks indicates that this method has good generalization and can be extended to more industrial scenarios. Extensive experiments on the Gas benchmark dataset and a real-world 5G enabled production line demonstrate that the proposed method achieves mean precisions of 97.35% and 97.41%, respectively. Compared with the non-vectorized data, the processing time for 5G data and EdgeIIoT data decreased by 79.4 s and 52.9 s, respectively. The ablation study confirms that incorporating the intention component (*T*) yields a 13.92-percentage-point F1-score improvement over the *E* + *A* configuration. Many experiments have demonstrated the effectiveness and good generalization ability of the method proposed in this paper, and it is more suitable for actual industrial scenarios.

Future research will focus on four directions: first, uncertainty-aware intention modeling using Bayesian neural networks to quantify inference confidence and flag ambiguous cases requiring operator review; second, cross-site validation across diverse production lines to assess generalizability to different sensor configurations and process types; third, explainability enhancement via attention mechanisms that directly map predictions to the underlying E,A,T components; fourth, online real-time deployment with streaming data processing, addressing privacy-preserving inference and edge computing constraints.

## Figures and Tables

**Table 1:** Summary of key limitations of representative studies.

Study	Method	Human-Factor Integration	Limitation
Li et al. [11]	Zonotopic detection	X	Assumes perfect model knowledge
Fritz et al. [12]	Discrete-event detection	X	Ignores continuous dynamics
Xu et al. [15]	Sketch-based DDoS detection	X	Cyber-only, no physical context
Takiddin et al. [17]	Graph neural network	X	Black-box, no interpretability
Han et al. [19]	BPSW feature selection	X	Semantic-opaque features

**Table 2:** Summary of principal mathematical symbols.

Symbol	Meaning	Domain/Codomain
*η*	Irrelevant or noisy data in industrial scenarios	Subset of raw data
*μ*	Observable and unobservable industrial data	Subset of raw data
*Z* = *E* × *A* × *T*	Industrial situation space lattice	Complete distributive lattice
π	Covering morphism	*Z* → *V*

**Table 3:** Morphism decomposition and industrial significance.

Morphism Direction	Morphism Formality	Step Description	Industrial Significance
Preprocessing: HCPS → IS	$\begin{array}{l} E=W_{E}\cdot x+b_{E}\\ A=W_{A}\cdot x+b_{A}\\ T=W_{T}\cdot x+b_{T}, \end{array}$ Among them, *W_T_*, *W_A_*, *W_E_* are submatrices, and *b_E_*, *b_A_*, *b_T_* are sub-offsets, which satisfy $A=\left[\begin{array}{l} W_{E}\\ W_{A}\\ W_{T} \end{array}\right],B=\left[\begin{array}{l} b_{E}\\ b_{A}\\ b_{T} \end{array}\right]$	1. Feature decoupling: Extracting <*E*, *A*, *T*>; 2. Dimension alignment $x\in R^{n}$ is the original data in SCPS, and the output dimension is *dim*(*E*) + *dim*(*A*) + *dim*(*T*).	Real-time generation of industrial situation triplets
Morphing: IS → V	$F\left(S\right)=\xi \left(E\right)⊗\psi \left(A\right)⊗\phi \left(T\right)$ where ξ ψ,ϕ, are component encoding functions, and ⊗ denotes the tensor product.	1. Component encoding: Encoding *E*, *A*, *T* separately; 2. Feature fusion: Capturing cross-component interaction features through the tensor product.	Supports machine learning model input
Postprocessing: V → HCPS	$T\left(v\right)=A^{-1}\left(v-B\right)$	1. Decoupled vector features: $v=G\left(S\right)$ containing the fused information of <*E*, *A*, *T*>; 2. Inverse transformation calculation: Transforming to SCPS data through *A*^−1^.	Closed-loop control and physical feedback

**Table 4:** ISA-95 standard coverage rates.

ISA-95 Standard	Total Scenario Amount	Sampling Amount	Unique Scenarios	Duplicate Scenarios	Coverage Rate
L-0	30	20	20	0	100
L-1	30	20	20	0	99
L-2	30	20	19	1	100
L-3	30	20	20	0	100
L-4	30	20	19	1	99

**Table 5:** Statistical profile of 5G non-ferrous metal production dataset.

Sensor Type	Feature Dimension	Sampling Rate	Total Records	Normal Samples	Attack Samples
Temperature Sensor	1	10 Hz	22,215	12,647	9568
Triaxial Vibration	3	1 kHz	59,205	23,564	35,641
Current (3-phase)	3	500 Hz	37,397	2351	35,046
Acoustic Emission	1	2 kHz	39,276	26,614	12,662
5G Network Metrics	3	1 Hz	52,458	19,003	33,455
Total	11 dims	-	210,551	84,179	126,372

**Table 6:** Hyperparameter optimization.

Parameter	Type	Range	Optimal Parameter
Learning-rate	Real	(0.0001, 0.001)	0.001
NLSTM-units	Integer	(64, 256)	256
Dropout-rate	Real	(0.2, 0.5)	0.5
Conv-filters	Integer	(32, 128)	96

**Table 7:** Anomaly recognition performance of vectorized data in industrial situation.

Type	With Situation Data	With Situation Vector Data
Model	CNN-NLSTM	CNN-NLSTM
Elapsed Time	86 s	34 s
Accuracy	95.27	97.99
Loss	0.42	0.14
Recall	90.50	94.91
F1-Score	85.70	97.35

**Table 8:** Comparison of anomaly recognition performances of multiple categories.

Model	Data	Accuracy	Precision	Recall	F1-Score
XGBoost [27]	Vectorized Gas	82.37	91.45	87.22	89.29
LSTM-AE-OCSVM [28]	Vectorized Gas	94.63	85.71	89.04	87.34
OPDT [29]	Vectorized Gas	92.16	81.93	88.47	85.17
PEO-DBN [30]	Vectorized Gas	86.60	84.79	90.31	87.45
CNN-LSTM [31]	Vectorized Gas	94.12	97.56	91.08	94.22
CNN-NLSTM	Vectorized Gas	97.99	99.92	94.91	97.35
TCN	Vectorized Gas	90.18	86.64	90.59	88.57
Transformer	Vectorized Gas	88.75	93.24	94.84	94.03
Bi-LSTM	Vectorized Gas	83.76	93.41	82.92	87.89

**Table 9:** Comparison of performances of different types of attack detection.

Type	Accuracy	Precision	Recall	F1-Score
Normal	94.75	94.02	97.89	96
NMRI	96.85	92.48	0	0
CMRI	98.64	92.61	99.00	96
MSCI	99.85	95.71	88.00	92
MPCI	99.75	98.23	98.67	98
MFCI	99.64	96.46	0	0
DoS	98.87	74.76	53.69	62
Recon	99.68	95.78	100	98

**Table 10:** Ablation study on imbalance mitigation strategies.

Strategy	NMRI Recall	MFCI Recall	NMRI F1	MFCI F1
No balance	0	0	0	0
SMOTE	0	0	0	0
Focal Loss	74.1	68.5	75.1	61.9
SMOTE + Focal Loss	88.9	86.1	88.5	89.7

**Table 11:** Human factor ablation experiment.

Feature Tuple	F1-Score	Time/s	GPU Memory Usage (GB)
E (using only physical sensor data)	63.45	26	0.01
E-A (action sequence)	83.43	30	0.02
E-A-T (triplet data)	97.35	25	0.01

**Table 12:** Generalization experiments under 5G production-line data and EdgeIIoT.

Data	Accuracy	Precision	Recall	F1-Score	Time/s
Original 5G data	83.28	94.54	93.42	93.97	83
Vectorized 5G data	98.82	97.41	94.39	95.87	3.6
EdgeIIoT	88.73	84.9	70.18	76.79	62
Vectorized EdgeIIoT	96.51	94.86	95.96	95.41	5.1

## Data Availability

The data presented in this study are available on request from the corresponding author.
